# The Self-Regulation of Learning – Self-Report Scale for Sport Practice: Validation of an Italian Version for Football

**DOI:** 10.3389/fpsyg.2021.604852

**Published:** 2021-03-15

**Authors:** Eleonora Reverberi, Caterina Gozzoli, Chiara D’Angelo, Margherita Lanz, Angela Sorgente

**Affiliations:** ^1^Department of Psychology, Università Cattolica del Sacro Cuore, Milan, Italy; ^2^Department of Psychology, Università Cattolica del Sacro Cuore, Brescia, Italy

**Keywords:** young athletes, measurement invariance, validation, football, self-regulation of learning

## Abstract

Self-regulation of learning (SRL) is a key psychological factor that supports young athletes aiming to reach the elite level by promoting their involvement in deliberate practice. We contributed to the validation of the Italian version of the Bartulovic et al. (2017) Self-Regulation of Learning – Self-Report Scale for Sport Practice by testing its factorial structure, reliability, and measurement invariance among elite and non-elite football players, involving 415 male professional, semi-professional, and amateur youth academy players (*M*_age_ = 16.2, SD = 1.51). The original six-factor structure (planning, reflection, effort, self-efficacy, self-monitoring, and evaluation) did not fit the data well and a five-factor solution (where self-monitoring and evaluation items load on the same factor, named “self-supervision”) was a better fit. This five-factor solution was measurement invariant across groups of elite and non-elite athletes. We found that elite athletes scored significantly higher than non-elite ones in each SRL subprocess. Implications for future validation studies and for the use of this tool are discussed.

## Introduction

“*Obstacles don’t necessarily have to stop you. If you find yourself facing a wall, don’t go back and give up. Find out how to skip it, make a hole in it, or go around it.*”

–Michael Jordan

Michael Jordan, an uncontested basketball champion, used these words to underline how mindset makes the difference between a talented athlete and a “normal” one. In this work, we focused on self-regulation of learning (SRL) as an important skill of the athletic mindset (Chen and Singer, 1992; Cleary and Zimmerman, 2001; Burnette et al., 2013; Elbe and Wikman, 2017). SRL is the degree of engagement in one’s own learning process with the aim of improving and mastering a specific task (Zimmerman, 2006). In other words, a self-regulated athlete is able to set specific training goals (e.g., increase the precision of dribbling) and monitor improvements (e.g., counting the number and the accuracy of dribbles).

The cognitive, motivational, and emotional aspects of learning have been conceptualized in terms of the concept of self-regulated learning. Originally, scholars studied SRL as a personal disposition that affects individuals’ functioning across different domains (Zimmerman, 1986). However, when scholars came to understand that each domain implies specific characteristics of learning, the study of SRL was adapted to specific learning domains such as music (Varela et al., 2016), education (Nota et al., 2004), and sport (Cleary and Zimmerman, 2001; Larsen et al., 2012) above all (for a review see Burnette et al., 2013). Moreover, after initially being studied as a disposition (Zimmerman, 2002), SRL has come to be considered a skill that supports a learner in their engagement in a specific field of learning (Van de Wiel et al., 2004). This view of SRL as a skill that can be improved became widespread in the sport literature (Jonker et al., 2010; MacNamara et al., 2010; Larsen et al., 2012; Tedesqui and Young, 2015; Elbe and Wikman, 2017).

Studies have shown that SRL is important for young athletes since it motivates them to be willing to invest effort in deliberate practice (Tedesqui and Young, 2015) and competition (Toering et al., 2009; Bartulovic et al., 2017; McCardle et al., 2019). SRL appears to be crucial for young, talented athletes, as it allows them to benefit from practice by keeping them motivated and focused on the improvable aspects of their performance (Toering et al., 2009). The first tool developed to measure SRL was built to detect it as a stable learning disposition of a person in different domains, but it was not specifically developed for sport (Toering et al., 2012a). Bartulovic et al. (2017) developed the first sport-specific version of the tool based on Toering et al. (2009), while McCardle et al. (2018) extended the conceptual breadth of the subscales by adding items describing another important dimension linked to sport practice, *concentration.* Results of their studies show that SRL is a key process for maintaining practice regimens in young athletes and enabling them to constantly improve their sport abilities, building a sport specific tool able to discriminate among different athletic skill groups (i.e., regional, national, and international competition athletes).

We believe that possessing an easy-to-use tool to assess SRL as a skill in a specific sport could both help researchers deepen their knowledge about SRL and help practitioners that work with young high-level athletes to develop this skill. In this article, we have contributed to the validation of the Italian version of the Self-Regulation of Learning – Self-Report Scale (SRL-SRS) for Sport Practice (Bartulovic et al., 2017) by testing its factorial structure, its reliability and its measurement invariance among elite and non-elite athletes in the specific sport of football.

In the following paragraphs, we briefly describe the psychological factors involved in talent development in sport, with a specific focus on SRL and the evolution of the studies on this specific competence. After that, we review the literature regarding tools to assess SRL over the years, especially in sport. Finally, we present our adaptation of Bartulovic et al.’s (2017) SRL-SRS for Sport Practice in football and its main constituent factors.

### Psychological Factors Involved in Talent Development in Sport

Since the 1990s, scholars interested in the study of elite athletes have established the importance of developing specific psychological qualities from an early age (Gould et al., 1992, 1993; Thomas and Thomas, 1999). Consequently different authors have studied psychological factors involved in talent development, identifying many, including: motivation, self-confidence, sport intelligence, commitment, grit, goal setting, and self-talk, among others (Morris, 2000; Gould et al., 2002; Abbott and Collins, 2004; Young and Medic, 2008; MacNamara et al., 2010; Gledhill et al., 2017). Still today, no agreement has been reached for three main reasons. First, psychological factors that are important in talent development are likely to differ according to the specific sport (MacNamara et al., 2010). Second, in studying both youth and adult elite athletes, it is vital to decide which parameters will be used to define “elite” status, because the findings are related to the specific parameters used (see Swann et al., 2015). Third, these factors could be culturally specific and thus not comparable (Dohme et al., 2017).

Despite the lack of agreement, recent literature has distinguished two kinds of psychological factors implicated in sport performance (Dohme et al., 2017): psychological *characteristics* and psychological *skills*. Psychological characteristics refer to those qualities of the mind that are innate predispositions or traits (e.g., personality, sport intelligence, motivation, grit, and so on). Psychological characteristics enable effective talent development by allowing athletes to negotiate challenges (e.g., career transitions, recovery from injury, and change of team/coach), for example, helping them to stay committed to their sport despite difficulties (e.g., to handle school and high-level sport practice). Psychological skills are a set of learned abilities and competencies of the mind that allow specific results to be accomplished, or psychological characteristics to be regulated or developed (e.g., goal setting, reflection, and self-talk). The main difference between psychological characteristics and skills is that the former are dispositional while the latter are developed and context specific factors (Dohme et al., 2017). The debate about psychological characteristics and psychological skills also pertains to the SRL, as it was initially defined and studied as a psychological characteristic that emerged as a key factor in supporting athletes’ success (MacNamara et al., 2010; Toering et al., 2012b) but subsequently it was considered a sport specific-skill (McCardle et al., 2019). In other words, SRL was initially considered to be a psychological characteristic (Toering et al., 2012a), a personal disposition that the individual consequently presented in different life domains (e.g., education and sport). This idea has subsequently been criticized (Jonker et al., 2010; Toering et al., 2013), as scholars have preferred to consider the SRL a skill that can be developed in a specific sport context.

The SRL as conceptualized by Bartulovic et al. (2017) is a psychological skill specifically developed in each sport context. Consequently, our adaptation of the Bartulovic et al. (2017) scale to the Italian football context measures SRL as a psychological skill.

### Self-Regulation of Learning in Sport

Self-regulation of learning was initially studied in education (Räisänen et al., 2016; Fernandez-Rio et al., 2017); only later did scholars begin to study it in a sport context (Cleary et al., 2006; Elferink-Gemser and Hettinga, 2017; Erikstad et al., 2018). It was first defined as a set of self-generated thoughts, feelings, and behaviors that are planned and cyclically adapted based on performance feedback (Zimmerman, 1989). Subsequently, this definition was reformulated as “a set of metacognitive, motivational, and behavioral processes that interact to allow the learner to be proactive in the learning process” (Zimmerman, 2008, p. 705). In other words, SRL indicates the degree of engagement in one’s own learning process with the aim of improving and mastering a specific task. According to the social cognitive model proposed by Zimmerman (Zimmerman and Kitsantas, 1996; Kitsantas and Zimmerman, 1998), self-regulatory processes are connected to self-efficacy beliefs, attributions, and self-satisfaction reactions (for an overview, see Panadero, 2017).

In general, SRL is composed of two main processes: motivation and metacognitive strategies. Motivation concerns the intensity of a learner’s desire to pursue a specific goal. It is influenced by self-efficacy beliefs and determines the learner’s commitment to goals and performance, which is fundamental for engaging in self-regulatory strategies (Kitsantas and Kavussanu, 2011). On the other hand, metacognitive strategies concern the awareness of one’s learning goals and the ability to monitor the learning process and reflect on the adequacy of one’s learning level in order to adjust it if necessary (Zimmerman, 2006). Motivation and metacognitive strategies are themselves composed of different subprocesses.

Toering et al. (2012a) were the first to identify and study the six key SRL subprocesses—namely planning, self-monitoring, evaluation, reflection, effort, and self-efficacy—and develop a unique tool to assess SRL as a relatively stable attribute in multiple learning domains (i.e., sport, music, and school; Toering et al., 2012a). The first four subprocesses allow a learner to set specific goals of improvement, monitor execution, evaluate the effectiveness of the results, and reflect on how to eventually improve them. Effort describes the intensity of engagement in the execution of a task during training, while self-efficacy consists of the self-referred evaluation of competencies and skills. Overall, these subprocesses allow learners to understand which actions they should avoid because they are detrimental to their performance and which ones they should improve since they are particularly effective for their practice and performance.

After that first study, other scholars improved knowledge of SRL in the specific context of sport (Toering et al., 2013; Bartulovic et al., 2017; McCardle et al., 2018, 2019), although some issues remain unresolved (McCardle et al., 2019). First, there is a need to deepen understanding of the relative importance of each SRL constituent processes, their interactions, and their relation to underpinning knowledge. Secondly, there is a need to examine SRL with respect to the dimensions of generality-specificity, macro–micro levels, and aptitude-event measures. Third, there is a need to deepen knowledge of self-regulation of motivation and emotion in the sport practice context and fourth, the interplay between athletes’ self-processes and their environment needs to be explored further.

All in all, the most recent literature on SRL describes it as a sport-specific learning skill which allows young athletes to improve their performance more rapidly and achieve better outcomes, which in turn leads to a higher likelihood of being selected for the youth team of a professional football club and, consequently, increases the possibility of reaching an elite level (Toering et al., 2012b; Bartulovic et al., 2017; Gledhill et al., 2017; McCardle et al., 2018). Consequently, different tools for assessing SRL in sport have been developed in recent years.

### The Development of the Self-Regulation of Learning – Self-Report Scale for Sport Practice and Its Validations

In the last two decades, scholars have worked to develop a tool for measuring SRL (e.g., Kitsantas and Zimmerman, 2002; Cleary et al., 2006; Toering et al., 2009; Jonker et al., 2010; Bartulovic et al., 2017; Ikudome et al., 2017; McCardle et al., 2018). Below, we review the development of the SRL-SRS and its subsequent new versions and/or adaptations.

The Self-Regulation of Learning—Self-Report Scale (SRL-SRS) developed by Toering et al. [2012a, “Toering et al., (2012a) SRL-SRS” from now on] was the first questionnaire that has been developed and tested to detect SRL as a disposition of learners in many different domains of excellence (i.e., school, sport, and music), and its construct validity has been supported by different studies (e.g., Toering et al., 2012b; Pitkethly and Lau, 2016; Ikudome et al., 2017). Thus, the first version of the SRL-SRS measured SRL as a disposition which a person applied to more than one context of learning. Inspired by Zimmerman’s (2002) theory of self-regulated learning, Toering et al.’s (2012a) SRL-SRS measured SRL as a stable individual attribute, using six different subscales (planning, self-monitoring, evaluation, reflection, effort, and self-efficacy). Toering et al.’s (2012a) SRL-SRS was adapted from previous scales: the subscales of planning and effort were based on the self-regulatory inventory of Hong and O’Neil (2001), and the self-monitoring subscale was adopted from the Self-Regulation Trait Questionnaire of Herl et al. (1999). The evaluation items were adopted from the evaluation subscale of the Inventory of Metacognitive Self-Regulation (Howard et al., 2000), and the reflection subscale was based on the reflection subscale of the Reflective Learning Continuum (Peltier et al., 2006). Finally, self-efficacy was assessed with items based on the Generalized Self-efficacy Scale (Schwarzer and Jerusalem, 1995). This multidimensional scale was tested on two samples composed respectively of 601 and 600 Dutch adolescents aged 11–17 years. The findings suggested maintaining 46 items to measure the six SRL hypothesized subdimensions, yielding valid, and reliable scores (Toering et al., 2012a).

Subsequently, Pitkethly and Lau (2016) tested the 46-item Toering et al. (2012a) SRL-SRS in a Hong Kong Chinese adolescent population; the confirmatory factor analysis results suggested shortening the scale to a 32-item version, which maintained the six factors of the original model. This factor structure was also confirmed by a cross-validation analysis in a second sample of Hong Kong adolescents.

Ikudome et al. (2017) tested the 46-item Toering et al. (2012a) SRL-SRS on a sample of 508 Japanese university students belonging to different physical activity clubs (football, baseball, basket, dance, track and field, swimming, cycling, softball, badminton, ping pong, artistic and rhythmic gymnastics, and canoeing). They kept only 37 items that loaded on five factors: planning, self-monitoring, effort, self-efficacy, and evaluation/reflection. The authors found these five factors’ scores to be valid and reliable among different populations of learners.

Toering et al. (2013) were the first to develop a football-specific self-report questionnaire, as they realize it was necessary to have a self-report instrument measuring SRL as a disposition that players apply in the context of daily practice which can be used to monitor the extent to which football players take responsibility for their own learning. They developed a tool that included five factors, namely: reflection, evaluation, planning, speaking up, and coaching. Thus, scholars switched from a domain general disposition to more context-specific dispositions. Despite this change, the dispositional approach to SRL was disputed by subsequent scholars, as some of them considered SRL to be a sport-specific skill (Bartulovic et al., 2017) useful for discriminating among athletes at different competitive levels (McCardle et al., 2018).

Bartulovic et al. (2017) were the first to develop a version of the SRL-SRS that was designed to assess athletes’ responses in relation to sport practice tasks. This scale operationalized the SRL as a skill (rather than as a disposition). They called it the SRL-SRS for Sport Practice [“Bartulovic et al. (2017) SRL-SRS-SP” from now on]. They first vetted the SRL-SRS with an expert panel to confirm the face validity of items and to adapt them for sport practice. This process showed that many of the original items needed to be tailored to training (i.e., by adding “during practice”) and others were not relatable to athletes or had poor readability, and thus were rephrased or deleted. In this way, the authors showed that SRL needed to be framed as a sport specific skill and not as a general disposition. Subsequently, the authors tested the factorial validity of the resulting 48-item sport-training version on a sample of 272 North American athletes (73% males, *M*_age_ = 22.43, SD = 3.95), from different sports: athletics (e.g., cross country, track and field, and road running; 87%), swimming (8%), and other individual sports (e.g., nordic skiing and cycling). As the CFA did not confirm the original model, exploratory analyses were performed. The resulting tool had 31 items and six factors that could be equated to the Toering et al. (2012a) SRL-SRS.

Bartulovic et al. (2017) also assessed whether their scale was able to discriminate between elite and non-elite athletes. They found that both the total score of this scale and its six subscales’ scores were able to discriminate across different levels of competition (e.g., elite, less elite, and recreational). In particular, the total SRL score was higher in the elite group than in other groups. As regards the subscales, only self-monitoring predicted membership in the elite and less elite group compared to the recreationally competitive group, while planning, self-monitoring, effort, and self-efficacy separately predicted membership in the elite group compared to the other groups.

More recently, McCardle et al. (2018) examined Bartulovic et al.’s (2017) SRL-SRS-SP relating to sport practice while trying to reclaim items from Toering et al.’s (2012a) SRL-SRS and to expand the breadth of SRL. Analyses related to the expanded scale resulted in a panel of 53 items, including new items added by the authors. Original and refined models were tested on 482 Canadian athletes (*M* = 26.45, SD = 12.66; 55% females). These participants were individual (64%) or team athletes (36%) in: powerlifting (*n* = 119), volleyball (71), athletics (57), Olympic weightlifting (37), speed skating (34), swimming (32), basketball (22), curling (14), rugby (14), and 28 other sports (with 10 participants or fewer). McCardle et al.’s (2018) resulting psychometric analyses and skill group analyses (for criterion validity) suggested that a refined model was better. This model was represented by a survey with 26 items to measure five SRL processes: planning, checking (formerly self-monitoring), evaluating/reflection, effort (including concentration), and self-efficacy. As self-monitoring, evaluation, and reflection appeared to converge, showing too high correlations, the researchers merged evaluating and reflecting items in the same subscale. Also, this new version of the scale yielded scores that were able to discriminate international-level senior athletes from less skilled groups.

In the current article, we aimed to develop the Italian version of Bartulovic et al.’s (2017) SRL-SRS-SP in the specific sport of football and offer a first contribution to its Italian validation by collecting three different kinds of validity evidence: score structure validity, internal consistency, and measurement and structural invariance. In line with previous studies (Bartulovic et al., 2017; McCardle et al., 2018), we expect that players in higher-level clubs score higher in SRL, as it would support the hypothesis that more talented athletes have higher levels of this skill (Jonker et al., 2010). Bartulovic et al.’s (2017) SRL-SRS-SP was adopted instead of McCardle et al.’s (2018) because the latter scale was not yet available at the time of data collection.

## Materials and Methods

### Participants

The sample is composed of 415 young male football players from two professional (League A and B^1^, *N* = 127), two semi-professional (League C, *N* = 162), and four amateur (*N* = 128) youth academies of Italian football clubs. Participants were aged between 14 and 20 years (*M*_age_ = 16.2, SD = 1.51), and mainly lived in the north of Italy (91% were born in Italy, while 8.4% were foreign and among them 2.4% had dual nationality). Most players lived with their parents (87.6%), while a minority lived in a specific residential structure provided by the club (6.7%) or with one parent (4.3%). Most participants indicated that people in their family usually practice sport (75.4%) and one participant out of four (27%) indicated that in their family there was an athlete, specifically their father (12.4%), grandfathers (4.3%), siblings (6.2%), or cousins/aunts (10.3%). Half of the participants (55.3%) had played another sport in their life but have abandoned it, and on average, players have been playing soccer for 9.6 years (in their current club for 3.6 years). Finally, 59 players in the sample (14%) have played in the Youth National team of the respective category.

### Instrument

Each participant filled in a paper-and-pencil survey that, in addition to socio-demographic variables, included the 31-item Bartulovic et al. (2017) SRL-SRS-SP. This scale is composed of six subscales: planning (including 8 items, e.g., *“I determine how to approach a practice task before I begin”*); self-monitoring (including 4 items, e.g., *“I check aspects of my workout while doing it”*); evaluation (including 4 items, e.g., *“After finishing, I look back on the practice task to evaluate my performance”*); reflection (including 2 items, e.g., *“When thinking about my training, I often reflect on my strengths and weaknesses”*); effort (including 8 items, e.g., *“I keep working hard even when sport training tasks become difficult”*); self-efficacy (including 5 items, e.g., *“I know how to handle unforeseen situations during practice, because I am resourceful”*). As for the original version (Bartulovic et al., 2017), items 1–24 were scored on a five-point Likert-type scale that assessed how often specific situations occur during practice, ranging from (1) “Never” to (5) “Always,” while items 25–31 were scored on a 1–5 Likert scale that assessed how much the respondent agrees with each statement, ranging from “Not at all” to “Totally” (see Supplementary Material). To assure that the scale’s original meaning was respected, the scale was translated and back-translated by a sport psychologist and an English native speaker translator (Brislin, 1986).

### Procedure

After gaining approval from the Ethics Committee (number of protocol: 08/18) of the authors’ university, professional, semi-professional, and amateur football clubs’ managers were contacted via a formal call or through contacting players the authors knew. Authors used a convenience sampling strategy, since Football Clubs involved in the study were available to take part in the study having previous collaborated with some of the authors of the present work. Club managers collected the informed consent forms from adult players or parents of minor players. All participants agreed to take part in the research. After that, data collection was performed separately for each team. All participants were treated in accordance with the Declaration of Helsinki, and with the ethical guidelines for research provided by the Italian Psychological Association (Associazione Italiana di Psicologia, 2015).

### Data Analysis

Data analysis was performed in two steps. First, the factorial structure and internal consistency of the Bartulovic et al. (2017) SRL-SRS-SP scores were evaluated. Then, scale measurement and structural invariance of the scale were tested between elite and non-elite athletes. Elite and non-elite groups were defined according to the competitive level of their team (i.e., League A, B, and C teams were considered elite since players compete in the highest-level youth championship, while other teams were considered non-elite since they are amateur/recreational-level athletes). Both steps were performed using Mplus software (version 7.11) and adopting robust maximum likelihood (MLR) as the estimation method to address the items’ non-normal distribution (i.e., skewness and/or kurtosis higher than |1|; Muthen and Kaplan, 1985).

#### Factorial Structure and Reliability

Based on previous validation studies, a six-dimensional scale structure was expected. In order to test whether this factorial structure was valid for the Italian sample too, we performed a CFA and evaluated the goodness of fit of the model using the following indexes: χ2 value, root mean square error of approximation (RMSEA), standardized root mean square residual (SRMR), comparative fit index (CFI), and incremental fit index (IFI). A non-significant χ2 value indicates that the model is consistent with the data, even if it is strongly affected by sample size (Iacobucci, 2010). RMSEA and SRMR values below 0.05 and CFI values higher than 0.95 indicate good fit, while RMSEA and SRMR values below 0.08 and CFI values higher than 0.90 indicate sufficient model fit (Marsh et al., 2004). Finally, even though IFI is not a commonly used fit index (Brosseau-Liard and Savalei, 2014), it was adopted here because, unlike the CFI, it is not affected by the sample size (Bollen, 1989). As we have few subjects in relation to the number of parameters that needed to be estimated (above all for the measurement invariance models), it is important to judge the fit of models using a non-biased index. We manually calculated IFI using the formula reported by Brosseau-Liard and Savalei (2014), where values higher than 0.90 indicate sufficient model fit.

Once the factorial structure of the scale had been confirmed, the reliability of each factor was tested. As suggested by current guidelines (Dunn et al., 2014), internal consistency was estimated using the composite reliability (ω) instead of Cronbach’s alpha (α). To be sufficiently reliable, a subscale should have ω > 0.60 (Bagozzi and Youjae, 1988). For the “self-reflection” subscale that is composed of only two items, the classic methods used to estimate internal consistency (composite reliability and Cronbach’s alpha) are not adequate. As suggested by Eisinga et al. (2013), we evaluated its reliability by calculating the inter-item correlation using Spearman’s correlation. The scale is considered reliable when the inter-item correlation is included in the range from 0.15 to 0.50 (Clark and Watson, 1995).

#### Measurement and Structural Invariance

Using multi-group analysis, the measurement model of the Bartulovic et al. (2017) SRL-SRS-SP was compared across elite and non-elite athletes. In particular, four types of measurement invariance (configural, weak, strong, and strict invariance; see Widaman et al., 2014 for details) were tested, from weakest (configural) to strongest (strict invariance). To test whether a specific invariance level is achieved, the correspondent model is compared with the less constrained one. To perform this comparison, the ΔCFI was calculated, where a negative ΔCFI value lower than −0.010 indicates that the two compared models significantly differ from each other (Cheung and Rensvold, 2002). If a specific level of invariance is not met, partial invariance can then be tested to determine which parameters do not meet invariance across groups. Following Dimitrov’s (2010) suggestion, the path to start freeing parameters was selected based on the modification index in Mplus output, which for each parameter gives the expected drop in the model’s Chi-square value if this parameter is freely estimated.

Establishing measurement invariance across groups is necessary to infer that the observed test scores convey the same psychological meaning in the respective groups and allow cross-group comparisons to be made (Bowden et al., 2016). Indeed, only when measurement invariance is verified can any differences found across groups (e.g., factors having different levels of variability or means across groups) be interpreted as a real difference in the construct and not as a difference due to a measurement artifact. In particular, if weak measurement invariance is achieved (i.e., elite and non-elite group models have equivalent factor loadings), it is possible to compare the variability of each factor across groups. If strong measurement invariance is achieved (i.e., equivalent intercepts), it is possible to compare the total *latent* factor means across groups. Finally, if strict measurement invariance is achieved (i.e., equivalent residuals), it is also possible to compare the total *observed* scores of each subscale across groups (Dimitrov, 2010).

In order to determine whether the Bartulovic et al. (2017) SRL-SRS-SP latent factors’ variability were significantly different across elite and non-elite athletes, we respectively constrained each factor variance to be equivalent across groups and then verified whether these constraints substantially modified the model fit (ΔCFI < −0.010). Finally, to determine whether the six factors had different mean levels across groups, we respectively constrained each factor mean to be equivalent across groups. If these constraints significantly modified the model fit (ΔCFI < −0.010), we verified which factors’ means were significantly different across the two groups. As the first group’s (i.e., elite athletes) factor means needed to be constrained to zero to make the model converge, each factor mean that is significantly different from zero in the second group (i.e., non-elite athletes) indicates that this factor mean is significantly different across groups. These factor mean differences, being standardized, can be interpreted using the cut-off suggested by Cohen (1988): differences around 0.20 indicate “small” differences; differences of about 0.50 indicate “medium” differences; and differences greater than 0.80 indicate “large” differences.

All in all, six types of invariance tests were performed (configural, weak, strong, strict, factor variance, and factor mean invariance) for each group comparison. The first four types correspond to measurement invariance, while the last two types correspond to structural invariance (Widaman et al., 2014). While measurement invariance is designed to help establish equivalence/non-equivalence of score interpretations, structural invariance is designed to detect actual differences between groups in the variability or mean level of their scores.

## Results

Descriptive statistics for the 31 items of the Bartulovic et al. (2017) SRL-SRS-SP are reported in Table 1. As indicated in this table, for each item, we had a percentage of missing data ranging from 0 to 2.65% (404 respondents out of 415). These data were missing randomly [Little’s MCAR test (612) = 639.08; *p* = 0.217]. Missing data were managed by the full information maximum likelihood method in the following models.

**TABLE 1:** Descriptive statistics of the 31 items of the SRL-SRS for Sport Practice.

	***N***	**Min**	**Max**	***M***	**DS**	**Skewness**	**Kurtosis**
Item 1	414	1	5	3.80	1.01	−0.73	0.27
Item 2	414	1	5	4.20	0.84	−1.07	1.31
Item 3	413	1	5	3.89	0.83	−0.61	0.55
Item 4	414	1	5	3.94	0.93	−0.69	0.12
Item 5	415	1	5	3.49	1.03	−0.41	−0.23
Item 6	415	1	5	4.00	0.96	−0.86	0.49
Item 7	413	1	5	4.14	0.95	−1.11	1.01
Item 8	413	1	5	3.43	1.00	−0.29	−0.23
Item 9	411	1	5	4.06	0.96	−1.04	1.00
Item 10	412	1	5	3.14	1.20	−0.14	−0.80
Item 11	412	1	5	4.14	0.87	−0.84	0.33
Item 12	412	1	5	3.74	0.92	−0.46	−0.08
Item 13	410	1	5	4.07	0.96	−0.93	0.43
Item 14	413	1	5	3.69	0.97	−0.56	0.08
Item 15	410	1	5	4.01	0.88	−0.66	−0.01
Item 16	409	1	5	3.57	1.03	−0.52	−0.20
Item 17	413	1	5	3.76	0.94	−0.53	−0.19
Item 18	408	1	5	3.44	1.00	−0.32	−0.32
Item 19	410	1	5	3.74	1.07	−0.66	−0.11
Item 20	404	1	5	3.69	0.93	−0.46	−0.12
Item 21	409	1	5	3.86	1.01	−0.60	−0.38
Item 22	412	1	5	3.85	0.93	−0.66	0.20
Item 23	411	1	5	3.60	0.99	−0.46	−0.13
Item 24	412	1	5	3.86	1.07	−0.83	0.16
Item 25	412	1	5	3.49	0.90	−0.38	0.17
Item 26	412	1	5	3.61	0.90	−0.43	0.16
Item 27	412	1	5	3.70	0.95	−0.42	−0.35
Item 28	410	1	5	3.25	0.97	0.04	−0.41
Item 29	409	1	5	3.55	0.78	−0.09	0.12
Item 30	411	1	5	3.85	0.96	−0.60	−0.26
Item 31	410	1	5	3.63	0.89	−0.34	0.14

### Factorial Structure and Reliability

The CFA aiming to confirm the six-factor structure found by Bartulovic et al. (2017) showed good fit indices: χ^2^(419) = 647.61; *p* < 0.001; RMSEA = 0.036 (0.031–0.042); *p* = 1.000; CFI = 0.931; IFI = 0.972; SRMR = 0.052. Each item’s factor loading was between 0.23 (item 10 of the “self-monitoring” factor) and 0.78 (item 7 of the “effort” factor) and significant at *p* < 0.001. Correlations among the six factors were all significant at *p* < 0.001 and ranged from 0.51 (between self-efficacy and planning) to 0.98 (between self-monitoring and evaluation). We had some concerns about this correlation between self-monitoring and evaluation as it approached 1 (*r* = 0.982; *p* < 0.001), suggesting that these two factors measure the same facet of SRL.

Consequently, we decided to test an alternative five-factor model in which items belonging to self-monitoring and evaluation were loaded on the same latent factor, which we called “self-supervision.” This new factor combines two subprocesses of SRL that imply the ability to reflect on the actions carried out to solve exercises during (self-monitoring) and after (evaluation) training. Self-monitoring describes the ability to supervise own actions as they are performed, while evaluation allows the athlete to think back to what has been done in training to check the rightness.

Fit indices of this alternative model were very similar to the ones of the original model: χ^2^(424) = 650.48; *p* < 0.001; RMSEA = 0.036 (0.030–0.041); *p* = 1.000; CFI = 0.932; IFI = 0.973; SRMR = 0.052. In this measurement model, factor loadings ranged from 0.28 (item 10 of self-supervision scale) to 0.78 (item 7 of effort scale) and were all significant at *p* < 0.001, while correlations among factors ranged from 0.52 (between self-efficacy and planning) to 0.84 (between self-supervision and planning) and were all significant at *p* < 0.001. We provide details about factor loadings (see Table 2) and inter-factor correlations (see Table 3) of the six-factor and five-factor solution below. As reported in Table 2, after merging the “Self-Monitoring” and “Evaluation” factors, their items’ factor loadings remain pretty much the same as for the six-factor model, confirming that the content of these two factors was strongly equivalent.

**TABLE 2:** Standardized factor loading of the 31 items of the SRL-SRS for Sport Practice for the six-factor model and the five-factor model.

	**Six-factor model**	**Five-factor model**
	**Factor: planning**	**Factor: planning**

Item 1	0.553	0.552
Item 5	0.579	0.579
Item 8	0.590	0.590
Item 12	0.540	0.539
Item 14	0.612	0.612
Item 18	0.628	0.628
Item 20	0.553	0.553
Item 23	0.720	0.721

	**Factor: self-monitoring**	**Factor: self-supervision**

Item 3	0.653	0.652
Item 10	0.229	0.231
Item 16	0.607	0.610
Item 17	0.666	0.670

	**Factor: evaluation**	

Item 4	0.721	0.719
Item 19	0.651	0.637
Item 22	0.690	0.686
Item 24	0.611	0.600

	**Factor: effort**	**Factor: effort**

Item 2	0.768	0.769
Item 6	0.715	0.715
Item 7	0.782	0.782
Item 9	0.583	0.583
Item 11	0.554	0.554
Item 13	0.646	0.646
Item 15	0.780	0.779
Item 21	0.713	0.712

	**Factor: reflection**	**Factor: reflection**

Item 27	0.532	0.539
Item 30	0.616	0.609

	**Factor: self-efficacy**	**Factor: self-efficacy**

Item 25	0.735	0.737
Item 26	0.583	0.583
Item 28	0.372	0.371
Item 29	0.563	0.561
Item 31	0.587	0.586

**TABLE 3:** Correlations between factors in the six-factor and five-factor models.

**Correlations**	**Six-factor model**	**Five-factor model**
Planning with self-monitoring*	0.857	0.838
Planning with evaluation	0.819	/
Planning with effort	0.648	0.648
Planning with reflection	0.720	0.723
Planning with self-efficacy	0.515	0.516
Self-monitoring with evaluation	0.982	/
Self-monitoring* with effort	0.847	0.830
Self-monitoring* with reflection	0.764	0.803
Self-monitoring* with self-efficacy	0.627	0.612
Evaluation with effort	0.812	/
Evaluation with reflection	0.821	/
Evaluation with self-efficacy	0.596	/
Effort with reflection	0.698	0.698
Effort with self-efficacy	0.563	0.564
Reflection with self-efficacy	0.692	0.696

*In the five-factor model, “Self-Monitoring” should be read as “Self-Supervision,” that consists in integration of “Self-Monitoring” and “Evaluation” factors. This is why results referred to the evaluation factor are not reported in the five-factor model (using “/”).

Even if this alternative five-factor model is preferable to the original one given that it is more parsimonious (i.e., more degree of freedom), we estimated the reliability (internal consistency) of both factorial solutions in order to make our results more comparable with results of the previous validations (Table 4).

**TABLE 4:** Internal consistency of each factor within each measurement model.

**Subdimension**	**Six-factor model**	**Five-factor model**
Planning	0.83	0.83
Self-monitoring*	0.60	0.81
Evaluation	0.76	
Effort	0.88	0.88
Reflection**	0.34	0.34
Self-efficacy	0.70	0.70

*In the five-factor model, “Self-Monitoring” should be read as “Self-Supervision,” that consists in integration of “Self-Monitoring” and “Evaluation” factors. This is why results referred to the evaluation factor are not reported in the five-factor model (using “/”). **As this subscale is composed of only two items, its internal consistency has been estimated by Spearman correlation instead of composite reliability.

Within the six-factor model, the self-monitoring factor has just sufficient reliability (ω > 0.60; Bagozzi and Youjae, 1988). The relatively modest reliability is probably due to item 10 (“While I am engaged in a practice task, I know how much of it I still have to complete”), which has a low factor loading (0.23) with respect to other items of the Bartulovic et al. (2017) SRL-SRS-SP (factor loadings higher than 0.37). The five-factor model resolved this problem, as in this alternative model the self-supervision factor’s reliability is very good (0.81), probably because (1) the factor loading of item 10 increased from 0.23 to 0.28, and (2) the factor is composed of a higher number of items, which favors its internal consistency. All the other subscales were sufficiently reliable. In particular, planning, self-evaluation, effort, and self-efficacy had a composite reliability higher than 0.60 (Bagozzi and Youjae, 1988), while the two-item reflection subscale had an inter-item Spearman correlation within the acceptable range (Clark and Watson, 1995).

### Measurement and Structural Invariance

We first tested the measurement invariance of the Bartulovic et al. (2017) SRL-SRS-SP across elite and non-elite groups using the original six-factor model, but this model was problematic, as in the elite group the correlation between self-monitoring and evaluation was equivalent to 1, indicating a linear dependence between these two factors and not allowing the convergence of the model. This was further proof that the five-factor model should be preferred to the six-factor one, at the least for the Italian sample.

Consequently, the measurement and structural invariance tests were performed on the five-factor model. As reported in Table 5, this model fitted sufficiently well in both groups (i.e., configural invariance). RMSEA and SRMR values were more than satisfactory, while the CFI value was lower than the 0.90 cut-off. We did not interpret this as a sign of model misfit, as Lai and Green (2016) demonstrated that a disagreement between RMSEA and CFI (good RMSEA and not-sufficient CFI) is not an indicator of misfit but can depend on other factors such as having too few subjects with respect to the number of parameters to estimate. This is what happened in our case, as we had to estimate 206 parameters separately for the elite group (*n* = 288) and non-elite (*n* = 127) athletes. Indeed, IFI—which is not affected by sample size (Bollen, 1989)—had sufficient values (>0.90) in each model.

**TABLE 5:** Measurement and structural invariance of the SRL-SRS for Sport Practice among elite (*n* = 288) and non-elite (*n* = 127) athletes.

**Invariance**	**χ^2^**	***df***	***p***	**RMSEA (90% CI)**	**CFI**	**IFI**	**SRMR**	**ΔC***FI*
Configural	1286.97	848	<0.001	0.050 (0.044, 0.055)	0.876	0.921	0.065	
Weak	1305.82	874	<0.001	0.049 (0.043, 0.054)	0.878	0.924	0.069	+0.002
Strong	1334.21	900	<0.001	0.048 (0.043, 0.054)	0.878	0.923	0.070	0.000
Strict	1419.04	931	<0.001	0.050 (0.045, 0.055)	0.862	0.910	0.081	−0.016
– Freeing item 2	1401.98	930	<0.001	0.049 (0.044, 0.055)	0.867	0.914	0.079	−0.011
– Freeing item 7	1390.02	929	<0.001	0.049 (0.044, 0.054)	0.870	0.917	0.077	−0.008
Factor variance	1403.09	934	<0.001	0.049 (0.044, 0.054)	0.868	0.914	0.097	−0.002
Factor mean	1449.14	939	<0.001	0.051 (0.046, 0.056)	0.856	0.902	0.129	−0.012

As our CFI was influenced by the small sample size, we considered the five-factor model confirmed for both elite and non-elite athletes and we proceeded to test other levels of invariance.

Results (Table 5) showed that the 31 items of the Bartulovic et al. (2017) SRL-SRS-SP have equivalent (Δ_CFI_ > −0.010) factor loadings (weak invariance) and intercepts (strong invariance) across groups, allowing for latent factor variance and factor mean comparisons. When testing strict invariance (invariant residual variance), we found that two items had non-invariant residuals across groups. In particular, item 2 (“I put forth my best effort when performing tasks at practice”) and item 7 (“I don’t give up at practice even if a task is hard”) had lower residuals in the elite group (0.214 and 0.263, respectively) than in the non-elite group (0.451 and 0.529, respectively). According to Dimitrov (2010), it is not necessary for all items to be invariant; it is sufficient to have 80% fully invariant items to consider the scale sufficiently equivalent across groups. So, although the scale does not have full strict invariance, it is possible to compare observed mean scores across groups.

Finally, we tested the structural invariance of the Bartulovic et al. (2017) SRL-SRS-SP, comparing latent factors’ variability and mean across groups. When we constrained factors’ variance to be the same across elite and non-elite groups, the model fit did not change significantly (Δ_CFI_ > −0.010), indicating that the five factors have the same variance (i.e., variability) within the two groups. On the other hand, when we constrained the factors’ mean to be the same across the two groups, the model fit substantially decreased (Δ_CFI_ < −0.010), indicating that at least one factor had a significantly different mean level between elite and non-elite athletes. In order to identify means that were significantly different across groups, we tested which factors’ mean of the non-elite group was significantly different from zero (i.e., mean level of the elite group). Findings indicated that non-elite athletes had a significantly lower level of self-regulation than elite ones for all five factors of the scale: planning (−0.436; *p* = 0.001), self-supervision (−0.725; *p* < 0.001), self-reflection (−0.389; *p* = 0.012), effort (−0.810; *p* < 0.001), and self-efficacy (−0.330; *p* = 0.011). In sum, the Bartulovic et al. (2017) SRL-SRS-SP scores were comparable between elite and non-elite athletes (i.e., measurement invariance) and were sensitive to differences in factor means, as they were higher for the elite group. In particular, the five dimensions of self-regulation showed small differences (lower than 0.50) for planning, self-reflection, and self-efficacy scores, medium (between 0.50 and 0.80) differences for the self-supervision factor and strong (higher than 0.80) differences for the effort factor across the two groups.

## Discussion

Self-regulation of learning is the degree of an individual’s engagement in his/her own learning process, aimed at improving performance on a specific task (Zimmerman, 2006). In sport, it is currently considered one of the most important psychological skills that support the effective growth of young talented athletes (Elbe and Wikman, 2017), and is strongly linked to the specific context of learning (i.e., sport-specific, football-specific, and so on). In fact, research shows that SRL helps athletes to stay involved and motivated during the learning process (Toering et al., 2009; Jonker et al., 2010).

In the belief that possessing tools for measuring and monitoring SRL could help both researchers and applied sport psychologist practitioners, we developed the Italian version of the Bartulovic et al. (2017) SRL-SRS-SP and tested it on an Italian sample of football players of different competitive levels. In particular, we aimed to verify the score structure, reliability, and measurement invariance of the scale and compare players in order to test the tool ability to discriminate among competitive levels.

On testing the score structure, we found that the original six-factor solution was not confirmed in the Italian sample for three main reasons. First, self-monitoring and evaluation factors were too highly correlated; in the elite subgroup, this correlation was equal to 1, indicating that these two factors measure exactly the same aspect of self-regulation for elite athletes. Similar results of overcorrelations were also found in Ikudome et al. (2017) and McCardle et al.’s (2018) studies. In the former, the evaluation factor was merged with the reflection factor, while in the second study the authors obtained a correlation of 1 between evaluation and self-monitoring in testing the initial model. After a set of different analysis, McCardle et al. (2018) preferred to create a unique factor from evaluation and reflection, while the self-monitoring factor was renamed “checking” after adding some items to better distinguish this process from the others. Thus, our need to merge two factors of the scale can be considered in line with the literature. Second, in our six-factor model, item 10 (“While I am engaged in a practice task, I know how much of it I still have to complete”) had a low factor loading (0.23) that slightly improved (0.28) in the five-factor solution. Third, in the six-factor solution the self-monitoring factor yielded poor reliability scores (see Table 4), as already found in other studies (Pitkethly and Lau, 2016).

As an alternative solution, we proposed a five-factor model in which items from self-monitoring and evaluations loaded on the same factor, which we named “self-supervision.” We preferred this factorial solution over the original because it has higher factor loadings and yields five reliable factors. Furthermore, while the six-factor structure was not replicable in the elite subgroup (linear dependence between self-monitoring and evaluations), the five-factor structure was adequate for both subgroups (configural invariance) and was sufficiently invariant (more than 80% of items showing full strict invariance; Dimitrov, 2010) across the elite and non-elite athletes to allow meaningful comparisons of the five factor scores across groups.

Regarding the self-supervision factor that emerges from our analysis, it seems that young Italian football players have difficulties in distinguishing between the moment they monitor the execution of training tasks (self-monitoring) and the moment they rethink those tasks to evaluate their performance or eventually understand mistakes (evaluation). This can be due to a training methodology adopted by coaches that emphasizes the repetition of an action/exercise to solve training exercises, instead of leaving a player free to choose a strategy involving performing an exercise and reflecting on his level of performance. Recently, research on talent development has increasingly underlined the importance of adopting a holistic ecological approach in supporting talent development in youth sport (Vaeyens et al., 2009; Vaamonde and Villanueva, 2012; Larsen et al., 2013; Reverberi et al., 2020), as it helps athletes to develop a specific mindset in completing exercises rather than becoming a mere “executor,” resulting in more effective career development (i.e., low dropout and risk of overtraining, high engagement in sport, and more focus on improvement).

Therefore, the enhancement of SRL can be an important link between the work of the coach and the sport psychologist—or other professionals who work with young athletes—since it supports the skill to reflect on personal strengths and weaknesses, promoting the creation of individual improvement goals.

Overall, our study is the first to have analyzed the measurement and structural invariance of the Bartulovic et al. (2017) SRL-SRS-SP. Previous research has tested whether the SRL-SRS (Toering et al., 2009, 2012b), the Japanese SRL-SRS (Ikudome et al., 2017), and the SRL-SRS-SP (Bartulovic et al., 2017) were able to predict belonging to an elite or non-elite-level group, but these studies have not assessed whether the scale worked equivalently in the two groups. Our study is the first to question this equivalence. We found that only two items were non-invariant across groups (i.e., item 2 “I put forth my best effort when performing tasks at practice” and item 7 “I don’t give up at practice even if a task is hard,” both belonging to the effort factor); this means that for the non-elite group, the response that athletes give to those items depends on factors other than effort more than for elite athletes. To follow-up on this finding, it would be interesting to qualitatively investigate the meaning of effort for elite and non-elite athletes to enable a better understanding of how this important motivational aspect of self-regulation is interpreted by each group. With regard to structural invariance, our findings also show that the scale is able to identify differences across groups, i.e., non-elite athletes had a significantly lower level of self-regulation than elite ones for all five factors of the scale. This difference is in the expected direction, as it shows that young athletes placed in more competitive contexts are more able to benefit from learning in practice, which, if correctly supported, can lead them to advance their sporting career (i.e., attain professionalism or a senior level, according to the specific sport).

Often, sports agents that deal with the selection of young athletes for sports clubs and federations are not aware of this specific skill, even if they are able to recognize some of its features (i.e., greater effort during practice or the ability to constantly reflect on errors and improve). Coaches and agents may benefit from a tool to assess SRL improvement over time, to understand whether their training supported the development of such skill in their athletes. Importantly, when using this tool, coaches and agents have to be conscious of the limitations of self-report scales. As well documented by Young and Starkes (2006), self-report evaluation in sport could be affected by different biases such as social desirability or recall bias. We suggest that practitioners adopt this scale in conjunction with observational assessment and administer the scale right after the workout and/or each exercise, in order to reduce errors related to capacities for experience recall.

Verifying measurement and structural invariance is fundamental for both researchers and practitioners: the former can continue the study of the properties of the scale, creating a shortened version or investigating the difficulty in distinguishing monitoring and evaluation, while the latter can apply the present version of the scale with both elite and non-elite athletes to assess the initial level of the SRL and set up a plan of improvement of it.

Due to its explorative nature and convenience sampling procedure, the present study has some limitations. The first concerns the sample size, as it affected the fit indices of our models. At the same time, we intentionally adopted the IFI index which is not affected by sample size to partially overcome this limitation. Second, in the sample there are eight teams (four elite and four non-elite) of different numerical sizes: it would be important to collect data from more teams with strict homogeneity in the number of athletes to test the factorial structure of this five-factor solution using multi-level confirmatory factor analysis (MCFA; e.g., Margola et al., 2019). This would also allow possible variations in the SRL of athletes that pertain to the culture of the team (i.e., the methodology of training, the relationship with the coach, etc.) to be identified. Third, data have only been collected in football and from male players, reducing the generalizability of our findings. It would be necessary to expand the sample to include female athletes and athletes from other disciplines to test invariance across gender (i.e., male vs. female) and sports (e.g., individual, pair, or team sports). In other words, although our study seems to have found a reliable and valid scale structure, future studies are needed to overcome the limitations of the current study and to collect further evidence of validity. In particular, we think that future studies should: (1) collect data from larger sample and/or sample different for gender and sport; (2) test whether the five-factor score is replicable in other countries too; (3) assess whether item 10’s factor loading reaches a sufficient level (i.e., at least 0.30; Fabrigar et al., 1999) and, if not, evaluate the possibility of removing it from the scale; and (4) collect additional kinds of validity evidence (e.g., Sorgente and Lanz, 2019), such as criterion validity evidence, for example verifying if the SRL scores are able to predict the level of self-efficacy in sport practice (Costa et al., 2015) or sport performance (Robazza et al., 2009).

## Data Availability Statement

The raw data supporting the conclusions of this article will be made available by the authors, without undue reservation.

## Ethics Statement

The studies involving human participants were reviewed and approved by the Catholic University of Sacred Heart, Milan. Written informed consent to participate in this study was provided by the participants’ legal guardian/next of kin.

## Author Contributions

ER, CG, and CD’A conceived the study and collected the data. ER and AS ran the analysis and wrote the first draft of the manuscript. ML supervised the methodology and the data analysis of this work. All authors discussed the results and contributed to the final manuscript.

## Conflict of Interest

The authors declare that the research was conducted in the absence of any commercial or financial relationships that could be construed as a potential conflict of interest.

## Footnotes

1 League A and B are the two major level in the Italian Football Championship, while League C is a semi-professional one. Amateurs Clubs involved in the sample instead competed in minor regional or interregional championship.

## References

[B1] Abbott A. Collins D. (2004). Eliminating the dichotomy between theory and practice in talent identification and development: considering the role of psychology. *J. Sports Sci.* 22 395–408. 10.1080/02640410410001675324 15160593

[B2] Associazione Italiana di Psicologia (2015). Codice etico per la ricerca in psicologia. *Assoc. Ital. Psicol.* Available online at: www.aipass.org/node/11560 (accessed February 2, 2021).

[B3] Bagozzi R. P. Youjae Y (1988). On the evaluation of structural equation models. *J. Acad. Mark. Sci.* 16 74–94. 10.1177/009207038801600107

[B4] Bartulovic D. Young B. W. Baker J. (2017). Self-regulated learning predicts skill group differences in developing athletes. *Psychol. Sport Exerc.* 31 61–69. 10.1016/j.psychsport.2017.04.006

[B5] Bollen K. A. (1989). A new incremental fit index for general structural equation models. *Soc. Methods Res.* 17 303–316. 10.1177/0049124189017003004

[B6] Bowden S. C. Saklofske D. H. van de Vijver F. J. R. Sudarshan N. J. Eysenck S. B. G. (2016). Cross-cultural measurement invariance of the Eysenck personality questionnaire across 33 countries. *Pers. Individ. Dif.* 103 53–60. 10.1016/j.paid.2016.04.028

[B7] Brislin R. W. (1986). “The wording and translation of research instruments,” in *Field Methods in Educational Research*, eds Lonner W. J. Berry J. W. (Newbury Park, CA: Sage), 137–164.

[B8] Brosseau-Liard P. E. Savalei V. (2014). Adjusting incremental fit indices for nonnormality. *Multivariate Behav. Res.* 49 460–470. 10.1080/00273171.2014.933697 26732359

[B9] Burnette J. L. O’Boyle E. H. VanEpps E. M. Pollack J. M. Finkel E. J. (2013). Mind-sets matter: a meta-analytic review of implicit theories and self-regulation. *Psychol. Bull.* 139 655–701. 10.1037/a0029531 22866678

[B10] Chen D. Singer R. N. (1992). Self-regulation and cognitive strategies in sport participation. *Int. J. Sport Psychol.* 23 277–300.

[B11] Cheung G. W. Rensvold R. B. (2002). Evaluating goodness-of-fit indexes for testing measurement invariance. *Struct. Equ. Model.* 9 233–255. 10.1207/S15328007SEM0902_5 33486653

[B12] Clark L. A. Watson D. (1995). Constructing validity: basic issues in objective scale development. *Psychol. Assess.* 7 309–319. 10.1037/1040-3590.7.3.309 PMC6754793 30896212

[B13] Cleary T. J. Zimmerman B. J. (2001). Self-regulation differences during athletic practice by experts, non-experts, and novices. *J. Appl. Sport Psychol.* 13 185–206. 10.1080/104132001753149883

[B14] Cleary T. J. Zimmerman B. J. Keating T. (2006). Training physical education students to self-regulate during basketball free throw practice. *Res. Q. Exerc. Sport* 77 251–262. 10.5641/027013606X13080769704640 16898280

[B15] Cohen J. (1988). *Statistical Power Analysis for the Behavioral Sciences*, 2nd Edn. Hillsdale, NJ: Erlbaum.

[B16] Costa S. Polani D. Livi S. (2015). Una scala per la misura delle convinzioni di efficacia personale nel tennis. *G. Ital. Psicol. Sport* 24 3–8.

[B17] Dimitrov D. M. (2010). Testing for factorial invariance in the context of construct validation. *Meas. Eval. Couns. Dev.* 43 121–149. 10.1177/0748175610373459

[B18] Dohme L. C. Backhouse S. Piggott D. Morgan G. (2017). Categorising and defining popular psychological terms used within the youth athlete talent development literature: a systematic review. *Int. Rev. Sport Exerc. Psychol.* 10 134–163. 10.1080/1750984X.2016.1185451

[B19] Dunn T. J. Baguley T. Brunsden V. (2014). From alpha to omega: a practical solution to the pervasive problem of internal consistency estimation. *Br. J. Psychol.* 105 399–412. 10.1111/bjop.12046 24844115

[B20] Eisinga R. Te Grotenhuis M. Pelzer B. (2013). The reliability of a two-item scale: Pearson, Cronbach, or Spearman-Brown? *Int. J. Public Health* 58 637–642. 10.1007/s00038-012-0416-3 23089674

[B21] Elbe A. M. Wikman J. (2017). “Psychological factors in developing high performance athletes,” in *Routledge Handbook of Talent Identification and Development in Sport*, eds Baker J. Cobley S. Wattie N. (London: Routledge), 236–252.

[B22] Elferink-Gemser M. T. Hettinga F. J. (2017). Pacing and self-regulation: important skills for talent development in endurance sports. *Int. J. Sports Physiol. Perform.* 12 831–835. 10.1123/ijspp.2017-0080 28605209

[B23] Erikstad M. K. Høigaard R. Johansen B. T. Kandala N. B. Haugen T. (2018). Childhood football play and practice in relation to self-regulation and national team selection: a study of Norwegian elite youth players. *J. Sports Sci.* 36 2304–2310. 10.1080/02640414.2018.1449563 29521180

[B24] Fabrigar L. R. Wegener D. T. MacCallum R. C. Strahan E. J. (1999). Evaluating the use of exploratory factor analysis in psychological research. *Psychol. Methods* 4 272–299. 10.1037/1082-989x.4.3.272

[B25] Fernandez-Rio J. Cecchini J. A. Méndez-Gimenez A. Mendez-Alonso D. Prieto J. A. (2017). Self-regulation, cooperative learning, and academic self-efficacy: interactions to prevent school failure. *Front. Psychol.* 8:22. 10.3389/fpsyg.2017.00022 28154544 PMC5243853

[B26] Gledhill A. Harwood C. Forsdyke D. (2017). Psychosocial factors associated with talent development in football: a systematic review. *Psychol. Sport Exerc.* 31 93–112. 10.1016/j.psychsport.2017.04.002

[B27] Gould D. Dieffenbach K. Moffett A. (2002). Psychological characteristics and their development in Olympic champions. *J. Appl. Sport Psychol.* 14 172–204. 10.1080/10413200290103482

[B28] Gould D. Eklund R. C. Jackson S. A. (1992). 1988 U.S. Olympic wrestling excellence: I. Mental preparation, precompetitive cognition, and affect. *Sport Psychol.* 6 358–382. 10.1123/tsp.6.4.358

[B29] Gould D. Jackson S. A. Finch L. M. (1993). Life at the top: the experiences of U.S. national champion figure skaters. *Sport Psychol.* 7 354–374. 10.1123/tsp.7.4.354 8278672

[B30] Herl H. E. O’Neil H. F. Jr. Chung G. K. W. K. Bianchi C. Wang S. Mayer R. (1999). *Final Report for Validation of Problem-Solving Measures*, (CSE Technical Report No. 501). National Center for Research on Evaluation, Standards, and Student Testing website. Available online at: http://www.cse.ucla.edu/products/Reports/TECH501.pdf (accessed November 20, 2006).

[B31] Hong E. O’Neil H. F. Jr. (2001). Construct validation of a trait self-regulation model. *Int. J. Psychol.* 36 186–194. 10.1080/00207590042000146

[B32] Howard B. C. McGee S. Shia R. Hong N. S. (2000). Metacognitive self-regulation and problem-solving: expanding the theory base through factor analysis. *Paper Presented at the Annual Meeting of the American Educational Research Association*, New Orleans, LA.

[B33] Iacobucci D. (2010). Structural equation modeling: fit indices, sample size, and advanced topics. *J. Consum. Psychol.* 20 90–98. 10.1016/j.jcps.2009.09.003

[B34] IkudomeS.NakamotoH.MoriS.FujitaT. (2017). Development of the self-regulation of learning in sports scale. 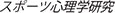 44 1–17.

[B35] Jonker L. Elferink-Gemser M. T. Visscher C. (2010). Differences in self-regulatory skills among talented athletes: the significance of competitive level and type of sport. *J. Sports Sci.* 28 901–908. 10.1080/02640411003797157 20544490

[B36] Kitsantas A. Kavussanu M. (2011). “Acquisition of sport knowledge and skill: the role of self-regulatory processes,” in *Handbook of Self-Regulation of Learning and Performance*, eds Zimmerman B. Schunk D. (London: Routledge) 217–233.

[B37] Kitsantas A. Zimmerman B. J. (1998). Self-regulation of motoric learning: a strategic cycle view. *J. Appl. Sport Psychol.* 10 220–239. 10.1080/10413209808406390

[B38] Kitsantas A. Zimmerman B. J. (2002). Comparing self-regulatory processes among novice, non-expert, and expert volleyball players: a microanalytic study. *J. Appl. Sport Psychol.* 14 91–105. 10.1080/10413200252907761

[B39] Lai K. Green S. B. (2016). The problem with having two watches: assessment of fit when RMSEA and CFI disagree. *Multivariate Behav. Res.* 51 220–239. 10.1080/00273171.2015.1134306 27014948

[B40] Larsen C. Alfermann D. Christensen M. (2012). Psychosocial skills in a youth soccer academy: a holistic ecological perspective. *Sport Sci. Rev.* XXI 51–74. 10.2478/v10237-012-0010-x

[B41] Larsen C. H. Alfermann D. Henriksen K. Christensen M. K. (2013). Successful talent development in soccer: the characteristics of the environment. *Sport Exerc. Perform. Psychol.* 2 190–206. 10.1037/a0031958

[B42] MacNamara Á Button A. Collins D. (2010). The role of psychological characteristics in facilitating the pathway to elite performance Part 1: identifying mental skills and behaviors. *Sport Psychol.* 24 52–73. 10.1080/03634520802237383

[B43] Margola D. Fenaroli V. Sorgente A. Lanz M. Costa G. (2019). The family relationships index (FRI): multilevel confirmatory factor analysis in an Italian community sample. *Eur. J. Psychol. Assess.* 35 335–345. 10.1027/1015-5759/a000427

[B44] Marsh H. W. Hau K. T. Wen Z. (2004). In search of golden rules: comment on hypothesis-testing approaches to setting cutoff values for fit indexes and dangers in overgeneralizing Hu and Bentler’s (1999) findings. *Struct. Equ. Model.* 11 320–341. 10.1207/s15328007sem1103_2

[B45] McCardle L. Young B. W. Baker J. (2018). Two-phase evaluation of the validity of a measure for self-regulated learning in sport practice. *Front. Psychol.* 9:2641. 10.3389/fpsyg.2018.02641 30627112 PMC6309132

[B46] McCardle L. Young B. W. Baker J. (2019). Self-regulated learning and expertise development in sport: current status, challenges, and future opportunities. *Int. Rev. Sport Exerc. Psychol.* 12 112–138. 10.1080/1750984X.2017.1381141

[B47] Morris T. (2000). Psychological characteristics and talent identification in soccer. *J. Sports Sci.* 18 715–726. 10.1080/02640410050120096 11043897

[B48] Muthen B. Kaplan D. (1985). A comparison of some methodologies for the factor analysis of non-normal Likert variables. *Br. J. Math. Stat. Psychol.* 38 171–189. 10.1111/j.2044-8317.1985.tb00832.x

[B49] Nota L. Soresi S. Zimmerman B. J. (2004). Self-regulation and academic achievement and resilience: a longitudinal study. *Int. J. Educ. Res.* 41 198–215. 10.1016/j.ijer.2005.07.001

[B50] Panadero E. (2017). A review of self-regulated learning: six models and four directions for research. *Front. Psychol.* 8:422. 10.3389/fpsyg.2017.00422 28503157 PMC5408091

[B51] Peltier J. W. Hay A. Drago W. (2006). Reflecting on reflection: scale extension and a comparison of undergraduate business students in the United States and the United Kingdom. *J. Mark. Educ.* 28 5–16. 10.1177/0273475305279658

[B52] Pitkethly A. J. Lau P. W. C. (2016). Reliability and validity of the short Hong Kong Chinese self-regulation of learning self-report scale (SRL-SRS-C). *Int. J. Sport Exerc. Psychol.* 14 210–226. 10.1080/1612197X.2015.1025810

[B53] Räisänen M. Postareff L. Lindblom-Ylänne S. (2016). University students’ self-and co-regulation of learning and processes of understanding: a person-oriented approach. *Learn. Individ. Dif.* 47 281–288. 10.1016/j.lindif.2016.01.006

[B54] Reverberi E. Angelo C. D. Littlewood M. A. Gozzoli C. F. (2020). Youth football players’ psychological well-being: the key role of relationships. *Front. Psychol.* 11:567776. 10.3389/fpsyg.2020.567776 33240153 PMC7683523

[B55] Robazza C. Bortoli L. Gramaccioni G. (2009). l’inventario psicologico della prestazione sportiva (IPPS-48). *G. Ital. Psicol. Sport* 4 14–20.

[B56] Schwarzer R. Jerusalem M. (1995). “Generalized self-efficacy scale,” in *Measures in Health Psychology: A User’s Portfolio. Causal and Control Beliefs*, eds Weinman J. Wright S. Johnston M. (Windsor: NFER-NELSON), 35–37.

[B57] Sorgente A. Lanz M. (2019). The multidimensional subjective financial well-being scale for emerging adults: development and validation studies. *Int. J. Behav. Dev.* 43 466–478. 10.1177/0165025419851859

[B58] Swann C. Moran A. Piggott D. (2015). Defining elite athletes: issues in the study of expert performance in sport psychology. *Psychol. Sport Exerc.* 16 3–14. 10.1016/j.psychsport.2014.07.004

[B59] Tedesqui R. A. B. Young B. W. (2015). Perspectives on active and inhibitive self-regulation relating to the deliberate practice activities of sport. *Talent Dev. Excell.* 7 29–39.

[B60] Thomas K. T. Thomas J. R. (1999). What squirrels in the trees predict about expert athletes. *Int. J. Sport Psychol.* 30 221–234.

[B61] Toering T. Elferink-Gemser M. T. Jonker L. van Heuvelen M. J. G. Visscher C. (2012a). Measuring self-regulation in a learning context: reliability and validity of the self-regulation of learning self-report scale (SRL-SRS). *Int. J. Sport Exerc. Psychol.* 10 24–38. 10.1080/1612197X.2012.645132

[B62] Toering T. Elferink-Gemser M. T. Jordet G. Visscher C. (2009). Self-regulation and performance level of elite and non-elite youth soccer players. *J. Sport Sci.* 27 1509–1517. 10.1080/02640410903369919 19967593

[B63] Toering T. Elferink-Gemser M. T. Jordet G. Visscher C. (2012b). Self-regulation of learning and performance level of elite young soccer players. *J. Sports Sci.* 43 312–325. 10.1080/02640410903369919 19967593

[B64] Toering T. Jordet G. Ripegutu A. (2013). Effective learning among elite football players: the development of a football-specific self-regulated learning questionnaire. *J. Sports Sci.* 31 1412–1420. 10.1080/02640414.2013.792949 23731271

[B65] Vaamonde A. G.-N. Villanueva P. J. (2012). Departamento de psicología del club Atlético de Madrid: filosofía, programación y desempeño profesional en el fútbol base. (Department of psychology of Club Atlético de Madrid: philosophy and performance management program in grassroots). *Cuad. Psicol. Deporte* 12 111–120. 10.4321/S1578-84232012000100010

[B66] Vaeyens R. Güllich A. Warr C. R. Philippaerts R. (2009). Talent identification and promotion programmes of Olympic athletes. *J. Sports Sci.* 27 1367–1380. 10.1080/02640410903110974 19787538

[B67] Van de Wiel M. W. Szegedi K. H. Weggeman M. C. (2004). “Professional learning: deliberate attempts at developing expertise,” in *Professional Learning: Gaps and Transitions on the Way from Novice to Expert*, eds Boshuizen H. P. A. Bromme R. Gruber H. (Dordrecht: Springer), 181–206. 10.1007/1-4020-2094-5_10

[B68] Varela W. Abrami P. C. Upitis R. (2016). Self-regulation and music learning: a systematic review. *Psychol. Music* 44 55–74. 10.1177/0305735614554639

[B69] Widaman K. F. Early D. R. Conger R. D. (2014). “Special populations,” in *The Oxford Handbook of Quantitative Methods: Foundations*, ed. Little T. D. (Oxford: Oxford University Press) 55–81.

[B70] Young B. W. Medic N. (2008). “The motivation to become an expert athlete: how coaches can promote long-term commitment,” in *Developing Elite Sports Performers: Lessons from Theory and Practice*, eds Farrow D. Baker J. MacMahon C. (New York, NY: Routledge) 43–59.

[B71] Young B. W. Starkes J. L. (2006). Measuring outcomes of swimmers’ non-regulation during practice: relationships between self-report, coaches’ judgments, and video-observation. *Int. J. Sports Sci. Coach.* 1 131–148. 10.1260/174795406777641320 29106335

[B72] Zimmerman B. J. (1986). Becoming a self-regulated learner: which are the key subprocesses? *Contemp. Educ. Psychol.* 11 307–313. 10.1016/0361-476X(86)90027-5

[B73] Zimmerman B. J. (1989). A social cognitive view of self-regulated academic learning. *J. Educ. Psychol.* 81 329–339. 10.1037/0022-0663.81.3.329

[B74] Zimmerman B. J. (2002). Becoming a self-regulated learner: an overview. *Theory Into Pract.* 41 64–70. 10.1207/s15430421tip4102

[B75] Zimmerman B. J. (2006). “Development and adaptation of expertise: the role of self-regulatory processes and beliefs,” in *The Cambridge Handbook of Expertise and Expert Performance*, eds Ericsson K. A. Charness N. Feltovich P. J. Hoffman R. R. (New York, NY: Cambridge University Press) 705–722. 10.1017/cbo9780511816796.039

[B76] Zimmerman B. J. (2008). Investigating self-regulation and motivation: historical background, methodological developments, and future prospects. *Am. Educ. Res. J.* 45 166–183. 10.3102/0002831207312909

[B77] Zimmerman B. J. Kitsantas A. (1996). Self-regulated learning of a motoric skill: the role of goal-setting and self-monitoring. *J. Appl. Sport Psychol.* 8 60–75. 10.1080/10413209608406308

