# Supplementary material for: The Self-Regulation of Learning – Self-Report Scale for Sport Practice: Validation of an Italian Version for Football
Source: Front Psychol. 2021 Mar 15;12:604852. doi: 10.3389/fpsyg.2021.604852 (PMC8005618; doi:10.3389/fpsyg.2021.604852)
Supplement: Supplementary file 1 [file Data_Sheet_1.PDF]

Supplementary Material

The Self-Regulation of Learning – Self-Report Scale (SRL-SRS) for Sport Practice: Validation of an Italian Version for Football

Original version

Read the following sentences and indicate which value best describes the way you behave when you meet a challenge, a difficulty, face a task or an exercise in training.
Think of a challenge or difficulty that you might encounter during training, or when you have to face a particularly difficult task in training. What do you do before facing it, while you do it and after doing it? And how often do you do these things when you compare yourself with a difficulty in training?

Remember, there are no right or wrong answers, simply describe yourself as you are, not how you would be or how you would act!

1<br>Never	2<br>Seldom	3<br>Sometimes	4<br>Often	5<br>Always

Italian version

Pensa a una sfida o una difficoltà che potresti incontrare durante l'allenamento, o a quando ti trovi a dover affrontare un compito particolarmente difficile in allenamento. Cosa fai prima di affrontarlo, mentre lo svolgi e dopo averlo svolto? E quanto spesso fai queste cose quando ti confronti con una difficoltà in allenamento?

Ricorda, non ci sono risposte giuste o sbagliate, semplicemente descriviti per come sei, non come vorresti essere o come vorresti agire!

1<br>Mai	2<br>Raramente	3<br>A volte	4<br>Spesso	5<br>Sempre

Item	Original version	Italian version	Factor
1	I determine how to approach a practice task before I begin.	Penso a come eseguire un compito prima di iniziarlo	Planning
2	I put forth my best effort when performing tasks at practice.	Do il mio massimo quando svolgo gli esercizi in allenamento	Effort
3	I check aspects of my workout while doing it.	Curo i vari aspetti dell'allenamento mentre lo faccio	Self-Monitoring*
4	I double-check to make sure I did practice tasks right.	Mi accerto di aver svolto gli esercizi correttamente	Evaluation*
5	I develop a plan for resolving difficulties at practice.	Sviluppo un piano per risolvere le situazioni di difficoltà in allenamento	Planning
6	Even when I don't like a task during practice, I work hard to do well.	In allenamento, anche se non mi piace un esercizio mi impegno al massimo per svolgerlo	Effort
7	I don't give up at practice even if a task is hard.	Continuo a lavorare duramente anche quando gli esercizi in allenamento sono difficili	Effort
8	Before I do a practice task, I think through the steps in my mind	Prima di eseguire dei compiti, pianifico le azioni che farò	Planning

9	I keep working hard even when sport training tasks become difficult.	In allenamento non mi arrendo anche se un esercizio è difficile	Effort
10	While I am engaged in a practice task, I know how much of it I still have to complete.	Mentre faccio un esercizio, so quanto mi manca ancora per finirlo	Self-Monitoring*
11	I am willing to do extra practice on tasks in order to acquire more skill.	Sono disposto ad allenarmi di più su alcuni compiti per diventare più capace	Effort
12	I try to understand the goal of a practice task before I do it.	Provo a capire l'obiettivo di un compito prima di eseguirlo	Planning
13	If I'm not really good at a task I can compensate for this by practicing hard.	Se non sono molto bravo in un esercizio, so di poter rimediare allenandomi più duramente	Effort
14	I ask myself questions about what a practice task requires me to do before I do it	Prima di svolgere un compito, penso a cosa mi richiede di fare	Planning
15	I work as hard as possible on all tasks at practice.	In allenamento, lavoro il più duramente possibile in tutti gli esercizi	Effort
16	I check my work all the way through a practice session	In allenamento, monitoro costantemente il mio lavoro	Self-Monitoring*
17	I check how well I am doing during practice tasks.	Mentre svolgo un compito, controllo quanto lo sto facendo bene	Self-Monitoring*
18	I clearly plan my course of action before starting practice tasks	Pianifico chiaramente le mie azioni prima di eseguire gli esercizi	Planning
19	After finishing, I look back on the practice task to evaluate my performance.	Dopo aver fatto un allenamento, ripenso se quello che ho fatto è giusto	Evaluation*
20	Before practice tasks, I figure out my goals and what I need to do to accomplish them.	Prima di svolgere gli esercizi, cerco di capire qual obiettivi hanno e cosa devo fare per raggiungerli	Planning
21	I work hard at practice on a task even if it is not important.	In allenamento mi impegno al massimo anche quando l'esercizio non è importante	Effort
22	I look back to see if I did the correct procedures at practice	Mi assicuro di aver svolto gli esercizi dell'allenamento correttamente	Evaluation*
23	Before practice tasks, I carefully plan my course of action	Prima di eseguire un compito, penso ai passaggi che lo compongono	Planning
24	I look back and check if what I did in practice was right.	Al termine dell'esercizio, valuto la mia prestazione	Evaluation*

Original version

Now indicate how much you agree with each of these phrases:

1<br>Not at all	2<br>Little	3<br>Enough	4<br>Much	5<br>Totally

Italian version

Ora indica quanto sei d'accordo con ognuna di queste frasi:

1<br>Per niente d'accordo	2<br>Poco d'accordo	3<br>Abbastanza d'accordo	4<br>Molto d'accordo	5<br>Totalmente d'accordo

25	I know how to handle unforeseen situations during practice, because I am resourceful	So gestire situazioni impreviste in allenamento, perché ho le risorse per farlo	Self-Efficacy
26	I am confident that I can deal efficiently with unexpected events at practice	Credo di poter affrontare in modo efficace gli eventi inaspettati in allenamento	Self-Efficacy
27	When facing difficulties at practice I can remain calm because I can rely on my coping abilities	Quando affronto delle difficoltà in allenamento riesco a rimanere tranquillo, perchè ho le capacità per affrontare la situazione	Reflection
28	When I am confronted with a difficulty during practice, I can usually find several solutions	Quando affronto momenti difficili durante l'allenamento, di solito riesco a trovare diverse soluzioni per risolverli	Self-Efficacy
29	No matter what comes my way at practice, I am usually able to handle it.	Non importa ciò che succede in allenamento perchè di solito sono in grado di gestirlo	Self-Efficacy
30	When thinking about my training, I often reflect about my strengths and weaknesses.	Quando penso ai miei allenamenti, spesso rifletto sui miei punti di forza e di debolezza	Reflection
31	I often think about my past experiences at practice to gain new insights	Spesso penso alle mie esperienze passate per avere nuove idee	Self-Efficacy

*In the Italian version, items originally belonging to Self-Monitoring and Evaluation load on the same factor, named "Self-supervision"
